# Complex Correntropy with Variable Center: Definition, Properties, and Application to Adaptive Filtering

**DOI:** 10.3390/e22010070

**Published:** 2020-01-06

**Authors:** Fei Dong, Guobing Qian, Shiyuan Wang

**Affiliations:** College of Electronic and Information Engineering, Chongqing Key Laboratory of Nonlinear Circuits and Intelligent Information Processing, Southwest University, Chongqing 400715, China; doufeiii@126.com (F.D.); wsy@swu.edu.cn (S.W.)

**Keywords:** complex, MCCC-VC, variable center, stability, EMSE

## Abstract

The complex correntropy has been successfully applied to complex domain adaptive filtering, and the corresponding maximum complex correntropy criterion (MCCC) algorithm has been proved to be robust to non-Gaussian noises. However, the kernel function of the complex correntropy is usually limited to a Gaussian function whose center is zero. In order to improve the performance of MCCC in a non-zero mean noise environment, we firstly define a complex correntropy with variable center and provide its probability explanation. Then, we propose a maximum complex correntropy criterion with variable center (MCCC-VC), and apply it to the complex domain adaptive filtering. Next, we use the gradient descent approach to search the minimum of the cost function. We also propose a feasible method to optimize the center and the kernel width of MCCC-VC. It is very important that we further provide the bound for the learning rate and derive the theoretical value of the steady-state excess mean square error (EMSE). Finally, we perform some simulations to show the validity of the theoretical steady-state EMSE and the better performance of MCCC-VC.

## 1. Introduction

Choosing the appropriate cost function (usually the statistical measure of error signal) is the key problem in adaptive filtering theory and application [1,2,3]. In the presence of Gaussian noise, it is best to using the minimum mean square error (MMSE) criterion. Therefore, a series of MMSE based algorithms [4,5,6,7] have emerged during the past decades. The MMSE based algorithms use the mean square value of the error between the desired signal and output signal as the cost function, which has many attractive features, such as convexity and smoothness. In addition, MMSE has low computational complexity since it only needs to calculate the second order statistics of the signals. However, in many non-Gaussian cases, the MMSE based algorithms are not robust. To improve this shortcoming, many kinds of non-MMSE criteria based algorithms have been developed in [8,9,10,11,12,13,14,15,16]. Since signals are often expressed in complex forms in many practical scenarios [17,18], adaptive filtering in complex domain is of great significance. During the past few years, some information criteria based algorithms have been proposed for complex domain adaptive filtering [19,20,21,22]. Particularly recently Guimarães defined a new similarity measurement between two complex variables based on complex correntropy [19,20] and proposed the maximum complex correntropy criterion (MCCC) algorithm. MCCC uses a complex Gaussian function as the kernel function, and derives the updation of weight based on Wirtinger Calculus. The complex Gaussian kernel function is desirable due to its smoothness and strict positive-definiteness. The performance of the MCCC algorithm is better than classic MMSE based algorithms, and is robust to non-Gaussian noise. Moreover, MCCC has been widely applied to many fields of machine learning and signal processing [23,24].

According to the MCCC, given two complex variables $C_{1}=A_{1}+jB_{1}$ and $C_{2}=A_{2}+jB_{2}$, complex correntropy is defined by [19,20]

(1) $$V_{\sigma }^{C}\left(C_{1},C_{2}\right)=E\left[\kappa \left(C_{1}-C_{2}\right)\right]$$

where $A_{1}$, $B_{1}$, $A_{2}$, $B_{2}$ represent real variables, E[⋅] denotes the expectation, and $\kappa \left(C_{1}-C_{2}\right)$ denotes the kernel function with

(2) $$\begin{array}{ll} \kappa \left(C_{1}-C_{2}\right) & =G_{\sigma }^{C}\left(C_{1}-C_{2}\right)\\ & =\frac{1}{2\pi \sigma^{2}}\exp\left(-\frac{\left(C_{1}-C_{2}\right){\left(C_{1}-C_{2}\right)}^{*}}{2\sigma^{2}}\right) \end{array}$$

and σ>0 is the kernel width.

The purpose of adaptive filtering is to estimate the target variable T in some sense by designing a model M to construct a output Y from input X. Under MCCC, we find this mode by maximizing the complex correntropy between T and Y:

(3) $$M^{*}=\underset{M\in ℳ}{\arg\max}V_{\sigma }^{C}\left(T,Y\right)=\underset{M\in ℳ}{\arg\max}E\left[G_{\sigma }^{C}\left(T-Y\right)\right]$$

where ℳ is the model assumption space which contains the possible models to construct the output Y from input X, and $M^{*}$ is the optimal model. 

However, the center of complex correntropy is always at zero, which is not the best option in the case of non-zero mean noise. Although the maximum corentropy criterion with variable center in [25] and [26] can be suitable for the variable center, they cannot be used for complex domain adaptive filtering. To overcome their defects, this paper proposes the maximum complex correntropy criterion with variable center (MCCC-VC).

The main contributions of this research lie in the following aspects: (1) we define a MCCC-VC and give its probability explanation; (2) based on the MCCC-VC, we propose a novel adaptive filtering algorithm in complex domain by utilizing the gradient descend approach; (3) we give effective and feasible methods to estimate the kernel center and update the kernel width adaptively; (4) we derive the bound for the learning rate, and the theoretical steady-state excess mean square error (EMSE) of the MCCC-VC algorithm, and verify the theoretical analysis by simulations.

The organization of this paper is as follows: Section 2 defines the complex correntropy with variable center and studies its properties. Section 3 proposes the MCCC-VC algorithm and provides the method for the optimization of the parameters. In addition, Section 3 also studies the convergence of the MCCC-VC algorithm and derives the theoretical steady-state excess mean square error (EMSE). Section 4 verifies the correctness of the theoretical conclusions and the superior performance of the MCCC-VC algorithm. Finally, Section 5 summarizes the conclusion of this paper.

## 2. Complex Correntropy with Variable Center 

For two complex variables, the target variable T and the output Y, the complex correntropy with variable center is defined as:

(4) $$V_{\sigma ,c}^{C}\left(T,Y\right)=E\left[G_{\sigma }^{C}\left(T-Y-c\right)\right]=E\left[\frac{1}{2\pi \sigma^{2}}\exp\left(-\frac{\left(T-Y-c\right){\left(T-Y-c\right)}^{*}}{2\sigma^{2}}\right)\right]$$

where c∈ℂ represents the center of the kernel function. When c=0, (4) will return to the original complex correntropy.

The complex correntropy with variable center c consists of the whole even moments of T−Y about the center c, which is as follows:

(5) $$V_{\sigma ,c}^{C}\left(T,Y\right)=\frac{1}{2\pi \sigma^{2}}\sum_{n=0}^{\infty }\frac{{\left(-1\right)}^{n}}{2^{n}n!}E\left[\frac{{\left|e-c\right|}^{2n}}{\sigma^{2n}}\right]$$

where e=T−Y is the complex valued error variable. With the increase of σ, the higher-order moments around the variable center c would attenuate quickly. Therefore, the second-order moment is the key factor which determines the value. In particular, when c=E[e] and σ→∞, maximizing the complex correntropy with c would be equal to minimizing the variance of the error.

Moreover, when σ→0, we obtain

(6) $$\begin{array}{ll} \underset{\sigma \to 0}{\lim}V_{\sigma ,c}^{C}\left(T-Y\right) & =\underset{\sigma \to 0}{\lim}\iint \iint G_{\sigma }^{C}\left(t_{R}-y_{R}-c_{R},t_{I}-y_{I}-c_{I}\right)p_{TY}\left(t_{R},t_{I},y_{R},y_{I}\right)dt_{R}dt_{I}dy_{R}dy_{I}\\ & =\iint \iint \delta \left(t_{R}-y_{R}-c_{R},t_{I}-y_{I}-c_{I}\right)p_{TY}\left(t_{R},t_{I},y_{R},y_{I}\right)dt_{R}dt_{I}dy_{R}dy_{I}\\ & =\iint p_{TY}\left(t_{R},t_{I},t_{R}-c_{R},t_{I}-c_{I}\right)dt_{R}dt_{I} \end{array}$$

where δ(x,y) is the two-dimensional Dirac function with $\left\{ \begin{array}{l} \iint \delta \left(x,y\right)dxdy=1\\ \delta \left(x,y\right)=0,\;\;\;\;\;\;x^{2}+y^{2}\neq 0 \end{array}\right.$, the second line is derived based on the fact that $\underset{\sigma \to 0}{\lim}G_{\sigma }^{C}\left(x,y\right)=\underset{\sigma \to 0}{\lim}\frac{1}{2\pi \sigma^{2}}\exp\left(-\frac{x^{2}+y^{2}}{2\sigma^{2}}\right)$ has the same property as δ(x,y), $t_{R}$, $y_{R}$, and $c_{R}$ are the real parts of t, y and c, $t_{I}$, $y_{I}$, and $c_{I}$ are the imaginary parts of t, y, and c, and $p_{TY}\left(t_{R},t_{I},y_{R},y_{I}\right)$ denotes the joint probability density function (PDF) of (T,Y). Furthermore, we derive the following result:

(7) $$\begin{array}{ll} \underset{\sigma \to 0}{\lim}V_{\sigma ,c}^{C}\left(T-Y\right) & =\underset{\sigma \to 0}{\lim}\iint G_{\sigma }^{C}\left(\varepsilon_{R}-c_{R},\varepsilon_{I}-c_{I}\right)p_{e}\left(\varepsilon_{R},\varepsilon_{I}\right)d\varepsilon_{R}d\varepsilon_{I}\\ & =\iint \delta \left(\varepsilon_{R}-c_{R},\varepsilon_{I}-c_{I}\right)p_{e}\left(\varepsilon_{R},\varepsilon_{I}\right)d\varepsilon_{R}d\varepsilon_{I}\\ & =\iint p_{e}\left(c_{R},c_{I}\right)d\varepsilon_{R}d\varepsilon_{I} \end{array}$$

where $p_{e}\left(\varepsilon_{R},\varepsilon_{I}\right)$ is the joint PDF of error. Thus, when σ→0, the value of complex correntropy with variable center c would approach $p_{e}\left(\varepsilon_{R},\varepsilon_{I}\right)$ evaluated at $\left(c_{R},c_{I}\right)$.

## 3. MCCC-VC Algorithm

In this part, we derive a novel adaptive filtering algorithm based on maximum complex correntropy criterion (i.e., minimum complex correntropy loss) with variable center (MCCC-VC).

### 3.1. Cost Function

We apply the MCCC-VC to adaptive filtering and derive the cost function as follows:

(8) $$\begin{array}{ll} J_{VC-loss}^{C} & =G_{\sigma }^{C}\left(0\right)-E\left[G_{\sigma }^{C}\left(e\left(k\right)-c\left(k\right)\right)\right]\\ & =\frac{1}{2\pi \sigma^{2}}\left\{1-E\left[\exp\left[-\frac{\left(\left(e\left(k\right)-c\left(k\right)\right){\left(e\left(k\right)-c\left(k\right)\right)}^{*}\right)}{2\sigma^{2}}\right]\right]\right\} \end{array}$$

where

(9) $$e\left(k\right)=d\left(k\right)-w^{H}x\left(k\right)$$

is the error at time instant k, $w={\left[w_{1}\;w_{2}\;\cdots \;w_{m}\right]}^{T}$ is the filter weight, d(k) is the desired signal at time instant k, $x\left(k\right)={\left[x\left(k\right)\;x\left(k-1\right)\;\cdots \;x\left(k-m+1\right)\right]}^{T}$ is the input signal at time instant k, c(k) is the center of the kernel at time instant k.

The essential idea behind the cost function (8) is that, in the practical case, even when the error distribution is non-zero-mean, the proposed MCCC-VC can perform well, because MCCC-VC matches well the error distribution.

**Figure 1:**
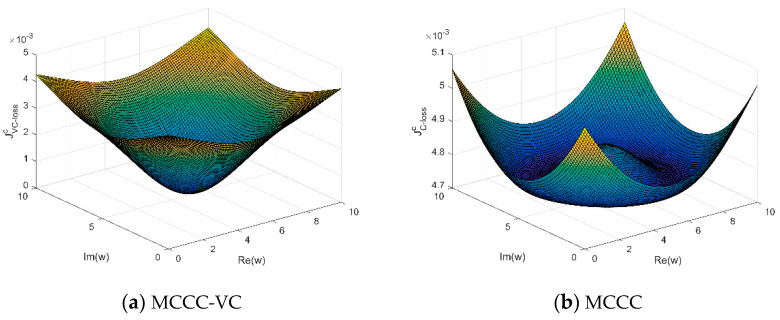
Surfaces of maximum complex correntropy criterion with variable center (MCCC-VC) and MCCC.

Figure 1 compares the surfaces of the proposed MCCC-VC with MCCC, where the noise is non-zero-mean complex Gaussian noise with unit variance. For visualization, we chose m=1, and set the system parameter and the mean of the noise as $w_{0}^{}=5+5i$ and c=6+6i, respectively. One can see that the cost function of MCCC-VC is minimized at $w_{0}^{}$, whereas the cost function of MCCC is minimized at some other place. 

### 3.2. Gradient Descent Algorithm Based On MCCC-VC

Since the stochastic gradient descent approach requires less computational complexity, we adopt it to search the minimum of the cost function. Utilizing Wirtinger Calculus [27,28], we obtain the updation of the weight as follows:

(10) $$\begin{array}{ll} w\left(k+1\right) & =w\left(k\right)-\mu \left\{\frac{\partial \left[1-\exp\left[-\frac{\left(e\left(k\right)-c\left(k\right)\right){\left(e\left(k\right)-c\left(k\right)\right)}^{*}}{2\sigma^{2}}\right]\right]}{\partial w^{*}\left(k\right)}\right\}\\ & =w\left(k\right)+\frac{\mu }{2\sigma^{2}}\exp\left[-\frac{{\left|e\left(k\right)-c\left(k\right)\right|}^{2}}{2\sigma^{2}}\right]{\left(e\left(k\right)-c\left(k\right)\right)}^{*}x\left(k\right)\\ & =w\left(k\right)+\eta_{w}\exp\left[-\frac{{\left|e\left(k\right)-c\left(k\right)\right|}^{2}}{2\sigma^{2}}\right]{\left(e\left(k\right)-c\left(k\right)\right)}^{*}x\left(k\right) \end{array}$$

where $\eta_{w}=\frac{\mu }{2\sigma^{2}}$ is the learning rate for the weight.

### 3.3. Optimization of the Parameters in MCCC-VC

#### 3.3.1. Optimization Problem in MCCC-VC

The two parameters center location c and the width of kernel σ act a pivotal part in the performance of MCCC-VC. Thus, it is extremely important to optimize them to further improve the robustness and convergence performance in the non-zero mean noise. 

The optimal model according to MCCC-VC is as follows:

(11) $$M*=\underset{M\in ℳ,\sigma \in \Omega ,c\in \mathbb{C}}{\arg\max}V_{\sigma ,c}^{C}\left(T,Y\right)=\underset{M\in ℳ,\sigma \in \Omega ,c\in \mathbb{C}}{\arg\max}E\left[G_{\sigma }^{C}(e-c)\right]$$

In addition, the complex correntropy with variable center can be divided into three parts:

(12) $$\begin{array}{ll} V_{\sigma ,c}^{C}\left(T,Y\right) & =\iint G_{\sigma }^{C}\left(\varepsilon_{R}-c_{R},\varepsilon_{I}-c_{I}\right)p_{e}\left(\varepsilon_{R},\varepsilon_{I}\right)d\varepsilon_{R}d\varepsilon_{I}\\ & =\frac{1}{2}{\iint \left[G_{\sigma }^{C}\left(\varepsilon_{R}-c_{R},\varepsilon_{I}-c_{I}\right)\right]}^{2}d\varepsilon_{R}d\varepsilon_{I}+\frac{1}{2}\iint {\left[p_{e}\left(\varepsilon_{R},\varepsilon_{I}\right)\right]}^{2}d\varepsilon_{R}d\varepsilon_{I}\\ & -\frac{1}{2}\iint \left[G_{\sigma }^{C}\left(\varepsilon_{R}-c_{R},\varepsilon_{I}-c_{I}\right)-p_{e}{\left(\varepsilon_{R},\varepsilon_{I}\right)}^{2}\right]d\varepsilon_{R}d\varepsilon_{I} \end{array}$$

Owing to the first term is independent from the optimal model, we can derive

(13) $$M*=\underset{M\in ℳ,\sigma \in \Omega ,c\in \mathbb{C}}{\arg\max}V_{\sigma ,c}^{C}\left(T,Y\right)=\underset{M\in ℳ,\sigma \in \Omega ,c\in \mathbb{C}}{\arg\max}U_{\sigma ,c}^{C}\left(T,Y\right)$$

where

(14) $$\begin{array}{ll} U_{\sigma ,c}^{C}\left(T,Y\right) & ={\iint \left[p_{e}(\varepsilon_{R},\varepsilon_{I})\right]}^{2}d\varepsilon_{R}d\varepsilon_{I}\\ & -{\iint \left[G_{\sigma }^{C}\left(\varepsilon_{R}-c_{R},\varepsilon_{I}-c_{I}\right)-p_{e}(\varepsilon_{R},\varepsilon_{I})\right]}^{2}d\varepsilon_{R}d\varepsilon_{I} \end{array}$$

and

(15) $$p_{e}\left(\varepsilon_{R},\varepsilon_{I}\right)=2E\left[G_{\sigma }^{C}(e_{R}-c_{R},e_{I}-c_{I})\right]$$

The parameters can be optimized by

(16) $$\left(M*,\sigma *,c*\right)=\underset{M\in ℳ,\sigma \in \Omega ,c\in \mathbb{C}}{\arg\max}U_{\sigma ,c}^{C}\left(T,Y\right)$$

where Ω and ℂ represent the allowed sets of parameters σ and c.

**Remark** **1.** *It can be seen that as long as the function* $U_{\sigma ,c}^{C}\left(T,Y\right)$ *is maximized,* M *,* σ *, and* c *can be optimized simultaneously. However, it is computationally demanding to compute and compare all the values of* $U_{\sigma ,c}^{C}\left(T,Y\right)$ *under all the possible parameters in the allowed sets. Moreover, it may be difficult to obtain the allowed sets of parameters.*

#### 3.3.2. Stochastic Gradient Descent Approach

To further simplify the optimization problem, we propose a stochastic gradient descent based online approach.

(1) When the model M is fixed, $\iint p_{e}(\varepsilon )d\varepsilon_{r}d\varepsilon_{I}$ is independent of the kernel width σ and the center position c. In this case, σ and c can be optimized according to the following formula:

(17) $$\begin{array}{ll} \left(\sigma *,c*\right) & =\underset{\sigma \in \Omega ,c\in \mathbb{C}}{\arg\min}{\iint \left[G_{\sigma }^{C}\left(\varepsilon_{R}-c_{R},\varepsilon_{I}-c_{I}\right)-p_{e}\left(\varepsilon_{R},\varepsilon_{I}\right)\right]}^{2}d\varepsilon_{R}d\varepsilon_{I}\\ & =\underset{\sigma \in \Omega ,c\in \mathbb{C}}{\arg\min}\left\{\iint {\left[G_{\sigma }^{C}\left(\varepsilon_{R}-c_{R},\varepsilon_{I}-c_{I}\right)\right]}^{2}d\varepsilon_{R}d\varepsilon_{I}-2E\left[G_{\sigma }^{C}(e-c)\right]\right\}\\ & =\underset{\sigma \in \Omega ,c\in \mathbb{C}}{\arg\min}\left\{-2E\left[G_{\sigma }^{C}(e-c)\right]+\frac{1}{4\pi \sigma^{2}}\right\} \end{array}$$

Provide *N* error samples ${\left\{e\left(k\right)\right\}}_{k=1}^{N}$, we can get $E\left[G_{\sigma }^{C}\left(e-c\right)\right]\approx \frac{1}{N}\sum_{k=1}^{N}G_{\sigma }^{C}\left(e\left(k\right)-c\left(k\right)\right)$. Therefore, we have the following formula:

(18) $$\left(\sigma *,c*\right)=\underset{\sigma \in \Omega ,c\in \mathbb{C}}{\arg\min}\left\{-\left[\frac{2}{N}\sum_{k=1}^{N}G_{\sigma }^{C}\left(e\left(k\right)-c\left(k\right)\right)\right]+\frac{1}{4\pi \sigma^{2}}\right\}$$

Furthermore, in order to simplify the optimization problem, we can set c(k) as the median or mean of the error samples. Thus, we only need to optimize σ. We take $1/\sigma^{2}$ as a new variable $\tilde{\sigma }$, and update $\tilde{\sigma }$ and $\sigma^{2}$ using the stochastic gradient descent approach as follows:

(19) $$\begin{array}{ll} \tilde{\sigma }\left(k+1\right) & =\tilde{\sigma }\left(k\right)-\eta_{\sigma }{\left[\frac{\partial -\left[\frac{2}{N}\sum_{l=k-T+1}^{k}G_{\sigma }^{C}\left(e\left(l\right)-c\left(k\right)\right)\right]+\frac{1}{4\pi \sigma^{2}}}{\partial \tilde{\sigma }}\right]|}_{\tilde{\sigma }=\tilde{\sigma }\left(k\right),c=c\left(k\right)}\\ & =\tilde{\sigma }\left(k\right)-\eta_{\sigma }\left\{-\left[\frac{1}{\pi N}\sum_{l=k-T+1}^{k}\exp\left(-\frac{{\left|e\left(l\right)-c\left(k\right)\right|}^{2}}{2}\tilde{\sigma }\left(k\right)\right)\left(1-\frac{{\left|e\left(l\right)-c\left(k\right)\right|}^{2}}{2}\tilde{\sigma }\left(k\right)\right)\right]+\frac{1}{4\pi }\right\} \end{array}$$

and

(20) $$\sigma^{2}\left(k+1\right)=\frac{1}{\tilde{\sigma }\left(k+1\right)}$$

where c(k) is estimated online as $c\left(k\right)=\sum_{l=k-T+1}^{k}e\left(l\right)$, and T is the smoothing length, $\eta_{\sigma }$ is the learning rate for $\tilde{\sigma }$.

(2) When the kernel width σ(k) and the center position c(k) is fixed, the model M is optimized by MCCC-VC using (10).

**Remark** **2.** *For the proposed MCCC-VC algorithm, the weight and the parameters are updated alternately at each time instant*k*using (10), (19) and (20), respectively*.

### 3.4. Performance Analysis

#### 3.4.1. Convergence Analysis

The MCCC-VC algorithm is written as a form of nonlinear error:

(21) $$w\left(k+1\right)=w\left(k\right)+\eta_{w}f\left(e\left(k\right)\right)x\left(k\right)$$

with $f\left(e\left(k\right)\right)=\exp\left[-\frac{{\left|e\left(k\right)-c\left(k\right)\right|}^{2}}{2\sigma^{2}}\right]{\left(e\left(k\right)-c\left(k\right)\right)}^{*}$ being the scalar function of the error e(k).

Taking into consideration that

(22) $$d\left(k\right)=w_{0}^{H}x\left(k\right)+v\left(k\right)$$

the error is written as

(23) $$e\left(k\right)={\tilde{w}}^{H}\left(k\right)x\left(k\right)+v\left(k\right)=e_{a}\left(k\right)+v\left(k\right)$$

where $\tilde{w}\left(k\right)=w_{0}-w\left(k\right)$ is the weight error vector at time instant k, $w_{0}^{}$ is the system parameter, $e_{a}\left(k\right)={\tilde{w}}^{H}\left(k\right)x\left(k\right)$ is the prior error, and v(k) is the additive noise at time instant k.

Therefore, we get the following formula

(24) $$\tilde{w}\left(k+1\right)=\tilde{w}\left(k\right)-\eta_{w}f\left(e\left(k\right)\right)x\left(k\right)$$

By taking the square of the 2-norm of both sides, we can further get the following formula:

(25) E{‖w˜(k+1)‖2}=E{‖w˜(k)‖2}−2ηwE{Re[ea(k)f(e(k))]}      +ηw2E{‖x(k)‖2|f(e(k))|2}

To guarantee the convergence of the MCCC-VC, the weight error power should be gradually decreased. Thus, we obtain the bound for the learning rate as follows:

(26) 0<ηw≤2E{Re[ea(k)f(e(k))]}E{‖x(k)‖2|f(e(k))|2}

#### 3.4.2. Steady-State Mean Square

If MCCC-VC arrives at steady-state, we have

(27) $$\underset{k\to \infty }{\lim}\;\;\;E\left\{{\left\|\tilde{w}\left(k+1\right)\right\|}^{2}\right\}=\underset{k\to \infty }{\lim}\;\;\;E\left\{{\left\|\tilde{w}\left(k\right)\right\|}^{2}\right\}$$

Then, when k→∞, we can get

(28) 2E{Re[(ea(k)−c)f(e(k))]}=ηwE{‖x(k)‖2|f(e(k))|2}

According to the definition of the steady-state excess mean square error (EMSE), we have

(29) $$S=\underset{k\to \infty }{\lim}\;\;\;E\left[{\left|e_{a}\left(k\right)\right|}^{2}\right]=E\left[{\left|e_{a}\right|}^{2}\right]$$

To obtain the theoretical steady-state EMSE, we present the following two assumptions [21,22,29]:

(1) v(k) is zero-mean distributed and independent of x(k), and x(k) is circular.
(2) $e_{a}\left(k\right)$ is zero-mean and independent of v(k).

Owing to the distributions of x(k), v(k), $e_{a}\left(k\right)$, and e(k) are not related to the time index k at the steady-state, the time index is ignored in the following derivation.

The left side of (28) can be written as

(30) $$\begin{array}{ll} L & =E\left\{e_{a}\left[\exp\left[-\frac{{\left|e-c\right|}^{2}}{2\sigma^{2}}\right]{\left(e-c\right)}^{*}\right]+e_{a}^{*}\left[\exp\left[-\frac{{\left|e-c\right|}^{2}}{2\sigma^{2}}\right]\left(e-c\right)\right]\right\}\\ & =E\left\{\exp\left[-\frac{{\left|e-c\right|}^{2}}{2\sigma^{2}}\right]\;\;\;\;\left(e_{a}{\left(e-c\right)}^{*}+e_{a}^{*}\left(e-c\right)\right)\right\}\\ & =E\left\{g_{1}\left(e\right)\left(2{\left|e_{a}\right|}^{2}+e_{a}v^{*}+e_{a}^{*}v\right)\right\} \end{array}$$

where

(31) $$g_{1}\left(e\right)=\exp\left[-\frac{{\left|e-c\right|}^{2}}{2\sigma^{2}}\right]\;\;\;\;$$

We use Taylor expansion to approximate $g_{1}\left(e\right)$ as

(32) g1(e)≈g1(v)+2Re{∂g1∂e|e=v⋅ea}+Re{∂2g1∂e*∂e*|e=v⋅(ea*)2+∂2g1∂e*∂e|e=v⋅|ea|2}

where

(33) $$\frac{\partial g_{1}}{\partial e}=\exp\left[-\frac{{\left|e-c\right|}^{2}}{2\sigma^{2}}\right]\;\times \left[-\frac{{\left|e-c\right|}^{2}}{2\sigma^{2}}{\left(e-c\right)}^{-1}\right]$$

(34) $$\frac{\partial g_{1}}{\partial e^{*}}=\exp\left[-\frac{{\left|e-c\right|}^{2}}{2\sigma^{2}}\right]\times \;\left[-\frac{{\left|e-c\right|}^{2}}{2\sigma^{2}}{\left({\left(e-c\right)}^{*}\right)}^{-1}\right]$$

(35) $$\frac{\partial^{2}g_{1}}{\partial e^{*}\partial e}=\exp\left[-\frac{{\left|e-c\right|}^{2}}{2\sigma^{2}}\right]\;\;\;\;{\left|e-c\right|}^{-2}\times \left[\frac{{\left|e-c\right|}^{4}}{{\left(2\sigma^{2}\right)}^{2}}-\frac{3{\left|e-c\right|}^{2}}{2\sigma^{2}}+\frac{{\left|e-c\right|}^{2}}{\sigma^{2}}\right]$$

(36) $$\frac{\partial^{2}g_{1}}{\partial e^{*}\partial e^{*}}=\exp\left[-\frac{{\left|e-c\right|}^{2}}{2\sigma^{2}}\right]\;\;\;\;{\left({\left(e-c\right)}^{*}\right)}^{-2}\times \left[\frac{{\left|e-c\right|}^{4}}{{\left(2\sigma^{2}\right)}^{2}}\right]$$

Owing to x is circular, we can get the values of the following two items:

(37) $$E\left[{\left(e_{a}^{*}\right)}^{2}\right]=0$$

(38) $$E\left[e_{a}{}^{2}\right]={\tilde{w}}^{H}xx^{T}{\tilde{w}}^{*}=0$$

Based on the above derivation, if the higher-order terms are small enough, we can rewrite the left side of (28) as follows:

(39) $$L\approx 2S\exp\left[-\frac{{\left|v\right|}^{2}}{2\sigma^{2}}\right]\;\;\;\;\times \left\{1-\frac{{\left|v\right|}^{2}}{2\sigma^{2}}\right\}$$

The right side of (28) can be written as

(40) $$\begin{array}{ll} R & =\eta_{w}Tr\left(R_{xx^{H}}\right)E\left\{{\left|f\left(e\left(k\right)\right)\right|}^{2}\right\}\\ & =\eta_{w}Tr\left(R_{xx^{H}}\right)E\left\{\exp\left[-\frac{{\left|e-c\right|}^{2}}{\sigma^{2}}\right]\;\;\;\;{\left|e-c\right|}^{2}\right\}\\ & =\eta_{w}Tr\left(R_{xx^{H}}\right)E\left\{g_{2}\left(e\right)\right\} \end{array}$$

where

(41) $$g_{2}\left(e\right)=\exp\left[-\frac{{\left|e-c\right|}^{2}}{\sigma^{2}}\right]\;\;\;\;{\left|e-c\right|}^{2}$$

In a similar way, we use a Taylor expansion to approximate $g_{2}\left(e\right)$ as

(42) g2(e)≈g2(v)+Re{∂2g2∂e*∂e|e=v⋅|ea|2+∂2g2∂e*∂e*|e=v⋅(ea*)2}+2Re{∂g2∂e|e=v⋅ea}

where

(43) $$\frac{\partial g_{2}}{\partial e}=\exp\left[-\frac{{\left|e-c\right|}^{2}}{\sigma^{2}}\right]\;\;\;\;{\left|e-c\right|}^{2}\times \left[-\frac{{\left|e-c\right|}^{2}}{\sigma^{2}}{\left({\left(e-c\right)}^{*}\right)}^{-1}+{\left(e^{*}\right)}^{-1}\right]$$

(44) $$\frac{\partial g_{2}}{\partial e^{*}}=\exp\left[-\frac{{\left|e-c\right|}^{2}}{\sigma^{2}}\right]\;\;\;\;{\left|e-c\right|}^{2}\times \left[-\frac{{\left|e-c\right|}^{2}}{\sigma^{2}}{\left({\left(e-c\right)}^{*}\right)}^{-1}+{\left(e^{*}\right)}^{-1}\right]$$

(45) $$\frac{\partial^{2}g_{2}}{\partial e^{*}\partial e}=\exp\left[-\frac{{\left|e-c\right|}^{2}}{\sigma^{2}}\right]\;\;\;\;\times \left[\frac{{\left|e-c\right|}^{4}}{\sigma^{4}}-\frac{3{\left|e-c\right|}^{2}}{\sigma^{2}}-1\right]$$

(46) $$\;\;\;\;\;\frac{\partial^{2}g_{2}}{\partial e^{*}\partial e^{*}}=\exp\left[-\frac{{\left|e-c\right|}^{2}}{2\sigma^{2}}\right]\;\;\;\;{\left|e-c\right|}^{2}{\left({\left(e-c\right)}^{*}\right)}^{-2}\left(\frac{{\left|e-c\right|}^{4}}{\sigma^{4}}-\frac{{\left|e-c\right|}^{2}}{2\sigma^{2}}\right)$$

If the higher-order terms are small enough, we can rewrite the right side of (28) as follows:

(47) $$R\approx \eta_{w}Tr\left(R_{xx^{H}}\right)E\left\{\exp\left[-\frac{{\left|v-c\right|}^{2}}{2\sigma^{2}}\right]\;\;\;\;{\left|v-c\right|}^{2}\right\}+\eta_{w}Tr\left(R_{xx^{H}}\right)S\;\;\times \;R_{1}$$

where

(48) $$R_{1}=E\{\exp\left[-\frac{{\left|v-c\right|}^{2}}{2\sigma^{2}}\right]\;\;\;\;\left(\frac{{\left|v-c\right|}^{4}}{\sigma^{4}}-\frac{3{\left|v-c\right|}^{2}}{\sigma^{2}}-1\right)\}$$

Finally, we get the theoretical steady-state EMSE as follows:

(49) $$S=\frac{\eta_{w}Tr\left(R_{xx^{H}}\right)E\left\{\exp\left[-\frac{{\left|v-c\right|}^{2}}{2\sigma^{2}}\right]\;\;\;\;{\left|v-c\right|}^{2}\right\}}{E\left\{2\exp\left[-\frac{{\left|v-c\right|}^{2}}{2\sigma^{2}}\right]\;\;\times \left[1-\frac{{\left|v-c\right|}^{2}}{2\sigma^{2}}\right]\right\}-\eta_{w}Tr\left(R_{xx^{H}}\right)R_{1}}$$

Furthermore, when $\eta_{w}$ is small enough, (49) is further simplified as

(50) $$S=\frac{\eta_{w}Tr\left(R_{xx^{H}}\right)E\left\{\exp\left[-\frac{{\left|v-c\right|}^{2}}{2\sigma^{2}}\right]\;\;\;\;{\left|v-c\right|}^{2}\right\}}{E\left\{2\exp\left[-\frac{{\left|v-c\right|}^{2}}{2\sigma^{2}}\right]\;\;\times \left[1-\frac{{\left|v-c\right|}^{2}}{2\sigma^{2}}\right]\right\}}$$

Moreover, we derive the theoretical value of $\sigma^{2}$ by setting $\frac{\partial \left\{-2E\left[G_{\sigma }^{C}(e-c)\right]+\frac{1}{4\pi \sigma^{2}}\right\}}{\partial \sigma^{2}}$=0. In this way, we have $\frac{1}{\pi \sigma^{4}}\exp\left(-\frac{{\left|e-c\right|}^{2}}{2\sigma^{2}}\right)-\frac{1}{\pi \sigma^{6}}\exp\left(-\frac{{\left|e-c\right|}^{2}}{2\sigma^{2}}\right)\frac{{\left|e-c\right|}^{2}}{2}-\frac{1}{4\pi \sigma^{4}}=0$. Due to e≈v at the steady state, we can further obtain the theoretical value of $\sigma^{2}$ based on the following approach:

(51) $$\sigma^{2}=\frac{E\left\{\frac{{\left|v-c\right|}^{2}}{2}\exp\left[-\frac{{\left|v-c\right|}^{2}}{2\sigma^{2}}\right]\right\}}{E\left\{-\frac{1}{4}+\exp\left[-\frac{{\left|v-c\right|}^{2}}{2\sigma^{2}}\right]\right\}}$$

Since the right side of (51) depends on $\sigma^{2}$, it is a fixed-point solution for the theoretical $\sigma^{2}$.

**Remark** **3.** *The theoretical steady-state EMSE in (50) is accurate only when* $e_{a}$ *is small enough, since the higher-order term can be negligible in this case. However, if the noise power or step size is too large, or the center position of the kernel function deviates from the mean of the noise, there will be a large deviation between the theoretical and simulated values of steady-state EMSE.*

## 4. Simulation

In this section, we present some simulations to show the validity of theoretical results and the superiority of MCCC-VC. We obtain all the simulation results by averaging over 300 Monte Carlo trials.

### 4.1. Steady-State Performance

In this part, the filter weight $w_{0}=[\begin{array}{lll} w_{1} & w_{2} & \cdots \end{array}{w_{10}]}^{T}$ is randomly generated, where $w_{k}=w_{Rk}+jw_{Ik}$, and $w_{Rk},w_{Ik}\in N\left(0,\;\;\;0.1\right)$, $w_{Rk}$ and $w_{Ik}$ represent the real and imaginary components of $w_{k}$, and $N\left(\mu ,\;\;\;{\hat{\sigma }}^{2}\right)$ denotes the Gaussian distributed variable whose mean and variance are μ and ${\hat{\sigma }}^{2}$, respectively. We randomly generate input signal $x=x_{R}+jx_{I}$. In order to show the robustness of MCCC-VC, additive complex noise $v=v_{R}+jv_{I}$ is added in the simulation, whose real and imaginary parts are denoted by $v_{R}$ and $v_{I}$, respectively. All algorithms initialize w with a zero vector.

**Figure 2:**
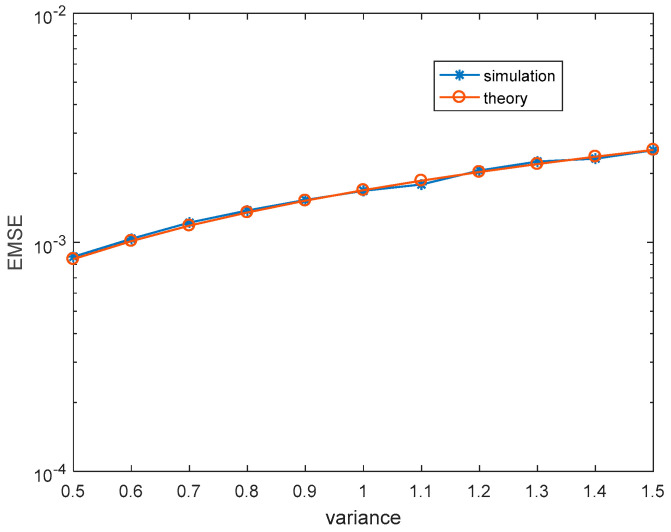
Steady-state excess mean square errors (EMSEs) under various $\sigma_{v}^{2}$. (Gaussian distributed noise,$\eta_{w}=3.8\times 10^{-4}$, $\eta_{\sigma }=4\times 10^{-3}$).

**Figure 3:**
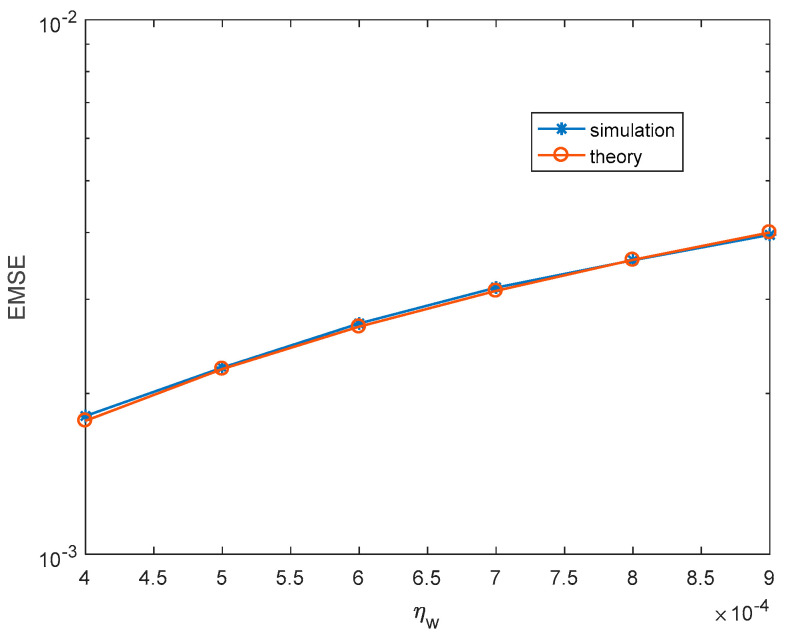
Steady-state EMSEs under various $\eta_{w}$. (Gaussian distributed noise, $\sigma_{v}^{2}=1$, $\eta_{\sigma }=4\times 10^{-3}$).

Firstly, we illustrate the correctness of theoretical steady-state EMSEs. For each simulation, 30,000 iterations are carried out to make sure MCCC-VC reaches the steady-state, and the last 1000 iterations are used to obtain the simulated steady-state EMSEs. The theoretical kernel width and steady-state EMSEs are calculated according to (51) and (50), respectively. Figure 2 and Figure 3 show the simulated and theoretical steady-state EMSEs of MCCC-VC under various noise variances and learning rates, where v is Gaussian distributed with mean 3+3j. It can be seen from both figures that theoretical results are closely matching with simulated results.

**Figure 4:**
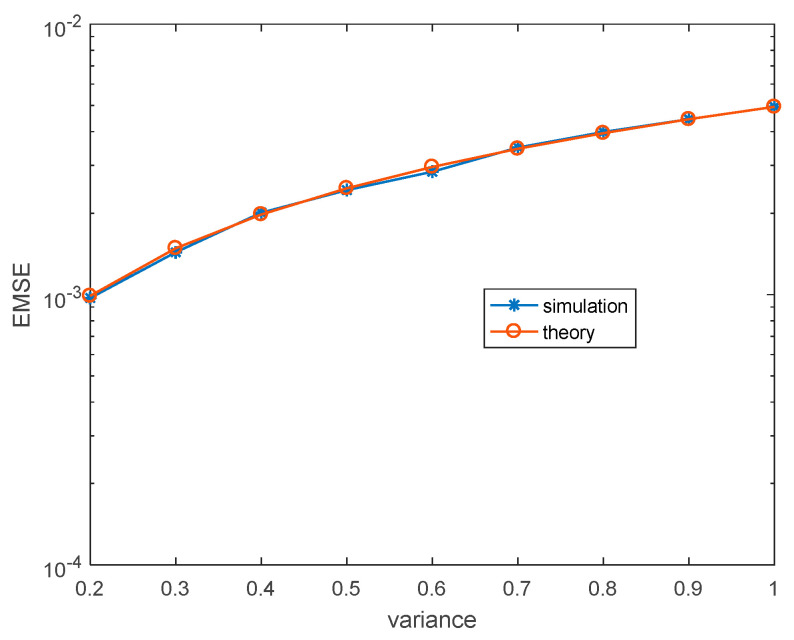
Steady-state EMSEs under various $\sigma_{v}^{2}$. (Binary distributed noise, $\eta_{w}=3.8\times 10^{-4}$, $\eta_{\sigma }=4\times 10^{-3}$).

**Figure 5:**
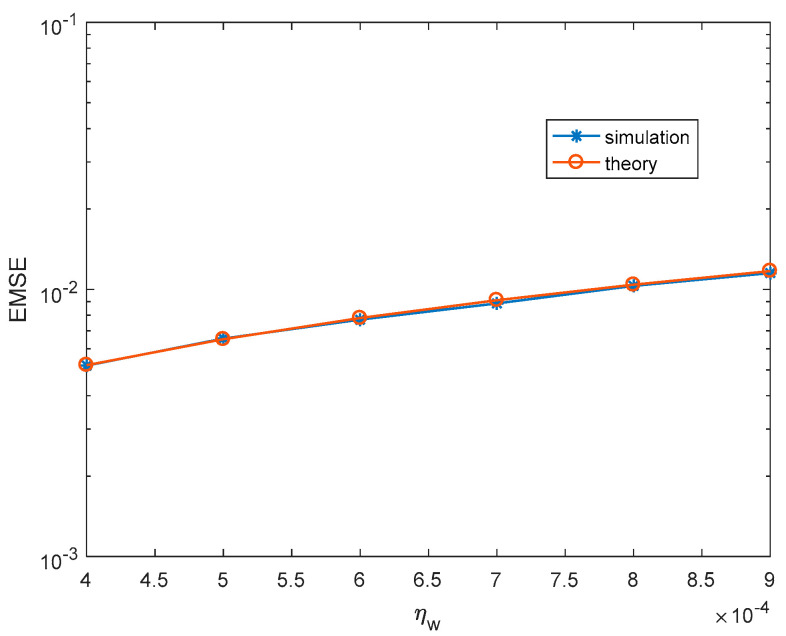
Steady-state EMSEs under various $\eta_{w}$. (Binary distributed noise, $\sigma_{v}^{2}=1$, $\eta_{\sigma }=4\times 10^{-3}$).

Then, we change the noise to binary noise, and the mean is also 3+3j. In addition, the simulated and theoretical steady-state EMSEs are obtained the same as before. Figure 4 and Figure 5 show the simulated and theoretical steady-state results of MCCC-VC under various noise variances and learning rates. Obviously, there is also a good matching between theoretical results and simulated results.

### 4.2. Performance Comparison

In this part, we compare the performance of the proposed MCCC-VC algorithm with MCCC and minimum complex kernel risk sensitive loss (MCKRSL) [22]. For the fair comparison, all three algorithms use the gradient descent method to search for the optimal solution. We measure the performance of all the algorithms by weight error power.

In this simulation, the noise v(k) is composed of two independent noises [16], i.e., v(k)=(1−a(k))A(k)+a(k)B(k), where P(a(k)=0)=1−c, and P(a(k)=1)=c (0≤c≤1). A(k) is the ordinary noise with small variance $\sigma_{v}^{2}=1$ whose real and imaginary parts are denoted by $A_{R}\left(k\right)$ and $A_{I}\left(k\right)$, and B(k) is the outliers with large variance whose real and imaginary parts are denoted by $B_{R}\left(k\right)$ and $B_{I}\left(k\right)$.

In this simulation, we set c=0.05 and $B_{R},\;\;B_{I}\in N\left(0,\;\;\;100\right)$. In addition, we consider the following four cases for A(k):

(1) $A_{R}\left(k\right),\;\;A_{I}\left(k\right)\in N\left(3,\;\;\;\sigma_{v}^{2}/2\right)$;
(2) $P\left(A_{R}\left(k\right)=3+\sigma_{v}^{}/\sqrt{2}\;\right)=P\left(A_{R}\left(k\right)=3-\sigma_{v}^{}/\sqrt{2}\right)=P\left(A_{I}\left(k\right)=3+\sigma_{v}^{}/\sqrt{2}\right)=P\left(A_{I}\left(k\right)=3-\sigma_{v}^{}/\sqrt{2}\right)=0.5$;
(3) $A_{R}\left(k\right),\;\;A_{I}\left(k\right)\in U\left(3-\sigma_{v}^{}/\sqrt{2},\;\;\;3+\sigma_{v}^{}/\sqrt{2}\right)$, with U(α,  β ) denoting the uniform distribution over [α,  β];
(4) $A_{R}\left(k\right)=3+\sigma_{v}^{}\sin\theta_{1k}/\sqrt{2}$, $A_{I}\left(k\right)=3+\sigma_{v}^{}\sin\theta_{2k}/\sqrt{2}$, where $\theta_{1k}\;,\theta_{2k}\in U[0,\;\;2\pi ]$.

**Figure 6:**
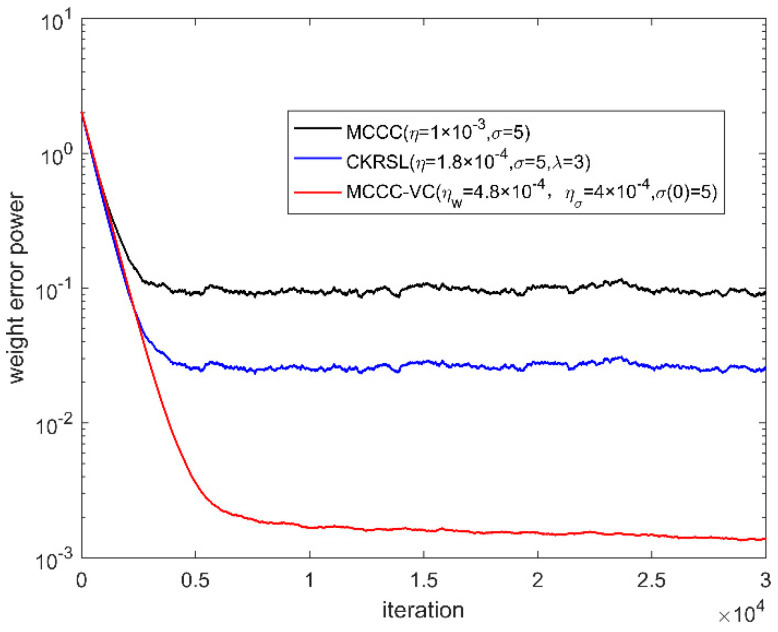
Convergence behavior of various algorithms (case 1).

**Figure 7:**
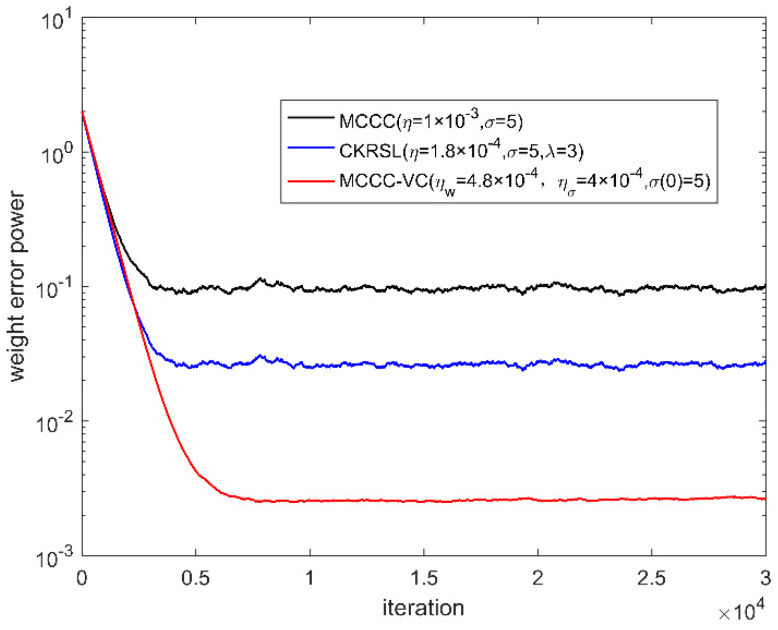
Convergence behavior of various algorithms (case 2).

**Figure 8:**
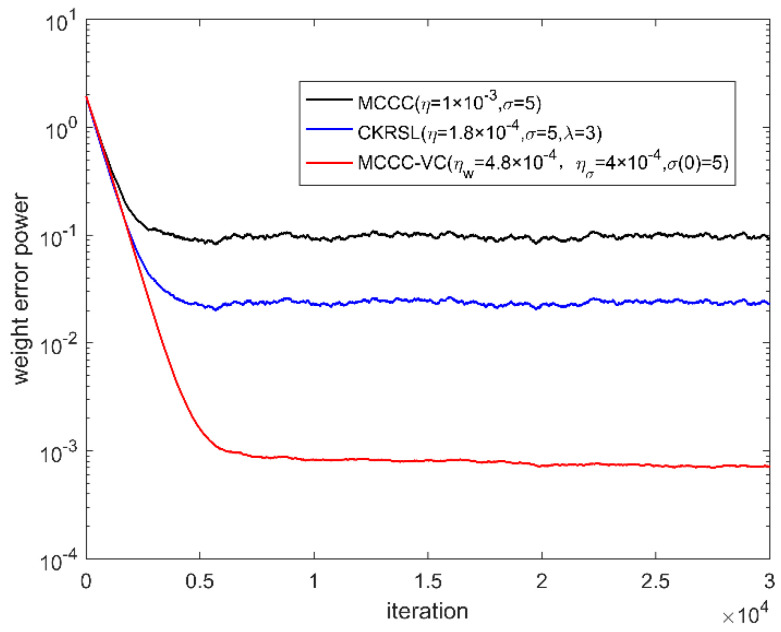
Convergence behavior of various algorithms (case 3).

**Figure 9:**
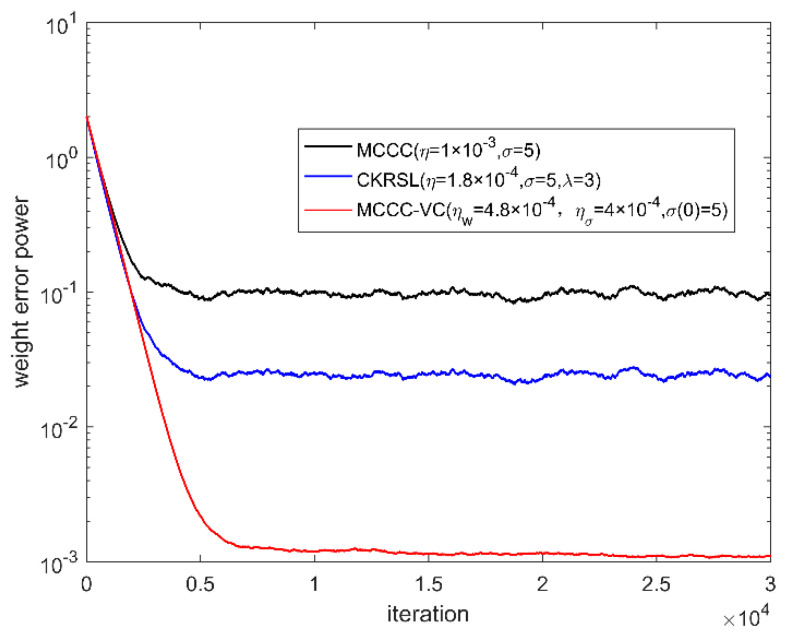
Convergence behavior of various algorithms (case 4).

Figure 6, Figure 7, Figure 8 and Figure 9 show the convergence behavior of various algorithms on the basis of weight error power ${\left\|w\left(k\right)-w_{0}\right\|}^{2}$ under different noises, where the parameter settings of different algorithms are summarized in Table 1. It can be seen clearly that the convergence performance of MCCC-VC is better than other two algorithms in all cases.

## 5. Conclusions

The complex correntropy usually employs a Gaussian kernel whose center is zero, which is not the best choice for many situations. To overcome this defect, this paper proposes the maximum complex correntropy criterion with variable center (MCCC-VC). The complex correntropy is extended to the case where the center can be anywhere. Furthermore, this paper also proposes an effective method to optimize the center position and the kernel width. More significantly, we analyze the convergence and steady-state performance of MCCC-VC theoretically. Simulation results obtained in Section 4 support the reliability of theoretical analysis and show the excellent performance of MCCC-VC.

## Figures and Tables

**Table 1:** Parameter setting of different algorithms.

Algorithm	MCCC	MCKRSL	MCCC-VC
Parameters	$\eta =1\times 10^{-3}$, σ=5.	$\eta =1.8\times 10^{-4}$, σ=5, λ=3.	$\eta_{w}=4.8\times 10^{-4}$, $\eta_{\sigma }=4\times 10^{-4}$, σ(0)=5.

Notes: *η* and σ denote the learning rate and kernel width for MCCC and MCKRSL, and *λ* denotes the risk-sensitive parameter for MCKRSL. Moreover, $\eta_{w}$, $\eta_{\sigma }$ denote the learning rates for the weight and kernel width of MCCC-VC, and σ(0) denotes the initial kernel with of MCCC-VC.
